# Widespread *Bathyarchaeia* encode a novel methyltransferase utilizing lignin‐derived aromatics

**DOI:** 10.1002/mlf2.12082

**Published:** 2023-09-18

**Authors:** Tiantian Yu, Haining Hu, Xianhong Zeng, Yinzhao Wang, Donald Pan, Longhui Deng, Lewen Liang, Jialin Hou, Fengping Wang

**Affiliations:** ^1^ School of Oceanography Shanghai Jiao Tong University Shanghai China; ^2^ State Key Laboratory of Microbial Metabolism, School of Life Sciences and Biotechnology Shanghai Jiao Tong University Shanghai China; ^3^ Southern Marine Science and Engineering Guangdong Laboratory (Zhuhai) Zhuhai China

**Keywords:** *Bathyarchaeia*, coastal sediments, lignin‐degrading, lignin‐derived aromatics, specific methyltransferase

## Abstract

Lignin degradation is a major process in the global carbon cycle across both terrestrial and marine ecosystems. *Bathyarchaeia*, which are among the most abundant microorganisms in marine sediment, have been proposed to mediate anaerobic lignin degradation. However, the mechanism of bathyarchaeial lignin degradation remains unclear. Here, we report an enrichment culture of *Bathyarchaeia*, named *Candidatus* Baizosediminiarchaeum ligniniphilus DL1YTT001 (*Ca*. B. ligniniphilus), from coastal sediments that can grow with lignin as the sole organic carbon source under mesophilic anoxic conditions. *Ca*. B. ligniniphilus possesses and highly expresses novel methyltransferase 1 (MT1, *mtgB*) for transferring methoxyl groups from lignin monomers to cob(I)alamin. MtgBs have no homology with known microbial methyltransferases and are present only in bathyarchaeial lineages. Heterologous expression of the *mtgB* gene confirmed *O*‐demethylation activity. The *mtgB* genes were identified in metagenomic data sets from a wide range of coastal sediments, and they were highly expressed in coastal sediments from the East China Sea. These findings suggest that *Bathyarchaeia*, capable of *O*‐demethylation via their novel and specific methyltransferases, are ubiquitous in coastal sediments.

## Impact statement

The members of *Bathyarchaeia* are among the most abundant microorganisms on the Earth and have long been considered key players in global carbon cycling. The lack of bathyarchaeial isolates until now largely hampers a direct understanding of the mechanisms of their carbon metabolism. Here, we present a successful continuous enrichment of *Bathyarchaeia* along with the discovery of a novel and specific methyltransferase for *O*‐demethylation from lignin‐derived aromatics. The key gene encoding this enzymatic system is widely present in genomes of *Bathyarchaeia* and in situ coastal sediments, indicating the important, yet unique role of *Bathyarchaeia* in lignin degradation. These findings shed light on the metabolism and evolution of lignin‐degrading microorganisms and provide insights into understanding microbially mediated carbon cycling in Earth environments.

## INTRODUCTION

As a complex aromatic polymer that is highly resistant to biological degradation, lignin comprises ~25% of the dry weight of vascular plants, which contributes 20%–50% of the organic carbon deposited in coastal sediments^1^, ^2^. Natural polymers of lignin are primarily composed of three aromatic monomers: *p*‐hydroxyphenyl (H), guaiacyl (G), and syringyl (S), of which the latter two groups are methoxylated aromatic compounds (ArOCH_3_), and 1.24%–24.1% of the carbon in lignin is in the form of methoxy groups^3^, ^4^. *O*‐Demethylation of the methoxy group, cleavage of the aromatic ring, and further degradation represent the main ligninolytic processes in anoxic environments because aerobic mechanisms of lignin depolymerization are not favorable in these environments^3^, ^5^.

Under anoxic conditions, acetogenic bacteria can demethylate from ArOCH_3_ and utilize the methyl groups as a source of energy by using bacterial‐type methyltransferase systems^6^. These bacteria include *Moorella thermoacetica* ^7^, *Acetobacterium dehalogenans* ^8^, *Sporomusa termitida* ^9^, and *Acetobacterium woodii* ^6^. In recent years, their methyltransferase systems were also detected in several archaeal groups, such as *Archaeoglobus fulgidus* ^10^ and a methanogenic archaeon *Methermicoccus shengliensis* ^11^. These bacteria and archaea acquired and shared bacterial‐type methyltransferase systems by horizontal gene transfer^10^. This methyltransferase system is cobalamin‐dependent demethylation and consists of four major enzymes, methyltransferase 1 (MT1), methyltransferase 2 (MT2), corrinoid protein (CP), and activating enzyme (AE), and the genes encoding these enzymes usually form gene clusters^8^, ^10^, ^11^, ^12^, ^13^. MT1 is the key enzyme responsible for substrate selectivity and determines the type of methyl substrate. MT1 transfers the cleaved methyl group to cob(I)alamin cofactor bound with CP, and MT2 catalyzes a subsequent transfer of the methyl group to tetrahydrofolate (THF) or to tetrahydromethanopterin (T_4_HMP). AE is required to reduce corrinoid in an ATP‐dependent reaction after inadvertent oxidation of the super‐reduced corrinoid into the inactive cob(II)alamin.

Members of the uncultured class *Bathyarchaeia* in the phylum *Thermoproteota* are among the most abundant microorganisms on the Earth^14^, ^15^ and are distributed in nearly all types of anoxic environments, particularly anoxic coastal sediments^15^, ^16^, ^17^. *Bathyarchaeia* have the genomic potential for diverse carbon metabolic processes and are thought to play an important role in the sediment carbon cycling^14^, ^15^, ^17^, ^18^, ^19^, ^20^. Our previous study found that the addition of kraft lignin to coastal sediments significantly stimulated the growth of *Bathyarchaeia* in anaerobic conditions^21^. During this process, assimilation of inorganic carbon into archaeal lipids was observed, which demonstrated an organoautotrophic lifestyle of *Bathyarchaeia* that utilizes lignin as an energy source and bicarbonate as a carbon source. These results indicated the important yet unrecognized role of *Bathyarchaeia* in anaerobic lignin degradation in sedimet.

However, the enzymatic system that mediates lignin degradation by this *Bathyarchaeia* remains unknown. No known genes encoding *O*‐demethylation or cleavage of the aromatic ring has been found in the metagenomic‐assembled genomes (MAGs) of this enriched *Bathyarchaeia* ^21^. In this study, we successfully cultured an archaeon of *Bathyarchaeia*, named *Candidatus* (*Ca*.) Baizosediminiarchaeum ligniniphilus DL1YTT001 (*Ca*. B. ligniniphilus). Combined with genomic, transcriptomic, proteomic, and enzymological analyses, we found that *Ca*. B. ligniniphilus use lignin‐derived ArOCH_3_ to grow and express a specific methyltransferase system that is distinct from the known methyltransferase system.

## RESULTS AND DISCUSSION

### Cultivation of an archaeon of *Bathyarchaeia*

**Figure 1:**
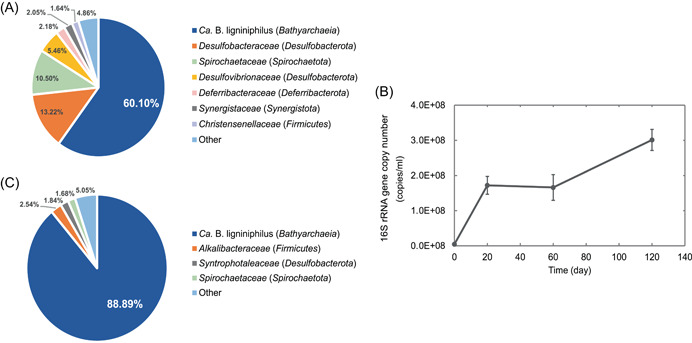
Microbial composition of enrichment cultures and growth curves of the cultured *Ca*. Baizosediminiarchaeum ligniniphilus. (A) Relative abundance of microbial populations based on the 16S rRNA gene‐tag sequencing analysis before purification by adding antibiotics. (B) Growth curves of *Ca*. B. ligniniphilus in anaerobic medium supplemented with lignin. The error bars were obtained from triplicate qPCR reactions. (C) Relative abundance of microbial populations based on the 16S rRNA gene‐tag sequencing analysis after purification by adding antibiotics.

**Figure 2:**
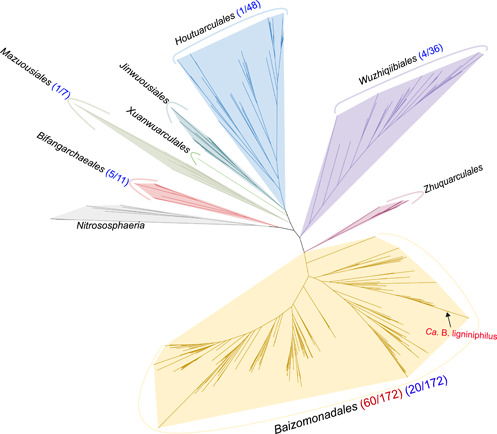
Maximum likelihood phylogeny of *Bathyarchaeia* based on concatenated alignments of 37 ribosomal proteins. Bootstrap values were calculated from 1000 iterations using IQ‐Tree. The numbers in parentheses represent the ratio of MAGs containing *Bathyarchaeia*‐specific *mtgB* genes (red) or bacterial‐type MT1 genes (blue) to total MAGs in each order. MAGs, metagenomic‐assembled genomes.

In 2015, we enriched *Bathyarchaeia* from coastal sediment samples by adding lignin as the sole organic carbon source, followed by 1‐year incubation^21^. The enrichment culture was transferred to artificial seawater medium supplemented with 5 g/l kraft lignin and 50 mM sodium bicarbonate; then, it was incubated at 35°C. The growth of *Bathyarchaeia* was confirmed by quantitative PCR (qPCR) after 2 months of incubation. Subsequently, the growth of *Bathyarchaeia* was maintained in our lab for more than 5 years by consecutive 1:10 transfers at 2–3‐month intervals. By December 2021, one archaeon of *Bathyarchaeia*, named *Ca*. B. ligniniphilus DL1YTT001, predominated in the enrichment culture, accounting for ~60% of the total abundance of 16S rRNA gene sequences (Figure 1A and Table S1). This enrichment culture contained additional groups primarily affiliated with *Desulfobacterota*, *Spirochaetota*, and *Deferribacterota*. The 16S rRNA gene copies of *Ca*. B. ligniniphilus increased 37‐fold from ~4.6 × 10^6^ to 1.7 × 10^8^ gene copies/ml in 20 days, which indicated a doubling time of ~3.8 days (Figure 1B). *Ca*. B. ligniniphilus was mesophilic, with an optimum growth temperature of 20°C (Figure S1); their cells appeared as small cocci with a diameter of ~500 nm and occurred individually or in chain‐like aggregates of several cells (Figure S2). Members of *Bathyarchaeia* were previously classified into 25 subgroups^17^, which are now assigned into eight orders^22^, ^23^. Phylogenetic inferences placed *Ca*. B. ligniniphilus in subgroup‐8 of the previous 16S‐based taxonomy for the phylum *Bathyarchaeota* ^17^ (Figure S3) and in the family *Baizostellaceae*, order *Baizomonadales*, of the newly proposed taxonomy for the class *Bathyarchaeia* based on 37 concatenated archaeal conserved proteins^20^, ^22^ (Figure 2).

To further purify *Ca*. B. ligniniphilus, the enrichment culture was supplemented with various bacteria‐specific antibiotics (ampicillin, vancomycin, kanamycin, and streptomycin, each at a concentration of 50 µg/ml) (Figure S4). The antibiotic cocktail not only efficiently inhibited bacterial growth but also suppressed the growth of *Ca*. B. ligniniphilus. Therefore, we hypothesize that the growth of *Ca*. B. ligniniphilus depends on metabolites produced by the cocultured bacteria. This dependence is illustrated by the lack of pathways in its MAG for the synthesis of some essential cofactors such as coenzyme F_420_ (Figure S5); however, more physical evidence will be needed to uncover this interaction mechanism.

The enrichment culture was transferred to fresh media supplemented with the aforementioned antibiotics as well as an additive of metabolites prepared from the supernatant of the same enrichment culture (Figure S4). After three transfers, *Ca*. B. ligniniphilus reached ~90% sequence abundance of all microbes in the enrichment (Figure 1C and Table S1), and a small fraction of sequences was primarily in the phyla of *Firmicutes, Desulfobacterota*, and *Spirochaetota*.

### Methyltransferase from methoxylated aromatic compounds (ArOCH_3_)

No known genes involved in ligninolytic processes (*O*‐demethylation of ArOCH_3_, cleavage of aromatic rings, or further degradation) were found in the genome of *Ca*. B. ligniniphilus (Table S2). To check for the occurrence of these processes during the incubation, metabolomes targeting low‐molecular‐weight (LMW) aromatic compounds on Day 0 and 30 of the incubation were analyzed using gas chromatography–mass spectrometry (GC‐MS). On Day 0, a variety of lignin‐derived ArOCH_3_, including guaiacol, vanillin, acetovanillone, vanillic acid, homovanillic acid, and syringic acid, were detected (Table 1). These monomers all dramatically decreased during the 30‐day incubation, and there was a substantial accumulation of their demethylated products, including catechol and protocatechoic acid, which confirmed the occurrence of O‐demethylation from ArOCH_3_ during the incubation of *Ca*. B. ligniniphilus.

**Table 1:** GC‐MS analysis of low‐molecular‐weight aromatic compounds in the *Ca*. B. ligniniphilus culture sampled at 0 and 30 days of the incubation.

		*Ca*. B. ligniniphilus‐1		*Ca*. B. ligniniphilus‐2		Medium‐1		Mediun‐2	
Retention (min)	Compound (μg/ml)	0 day	30 days	0 day	30 days	0 day	30 days	0 day	30 days
11.6	Guaiacol	1.0	0.0	0.8	0.0	0.9	0.8	1.0	1.1
18.29	Vanillin	6.6	0.2	2.5	0.1	5.1	4.7	7.3	6.4
19.92	Acetovanillone	6.9	0.0	6.2	0.0	7.0	6.9	7.4	7.0
22.61	Vanillic acid	12.6	0.0	11.2	0.0	12.1	12.8	12.9	12.8
22.68	Homovanillic acid	2.9	1.7	2.7	0.2	2.9	3.0	3.0	3.0
24.82	Syringic acid	1.4	1.4	1.4	0.0	1.5	1.5	1.4	1.5
13.62	Catechol	0.0	6.1	0.0	7.1	0.0	0.0	0.0	0.0
23.52	Protocatechoic acid	0.0	5.0	0.0	0.59	0.0	0.0	0.0	0.0

All assays were performed in duplicate. *Ca*. B. ligniniphilus‐1 and *Ca*. B. ligniniphilus‐2 were the *Ca*. B. ligniniphilus enrichment culture with lignin as the sole organic carbon source; medium‐1 and medium‐2 were the lignin media without inoculation and were used as negative controls.

**Figure 3:**
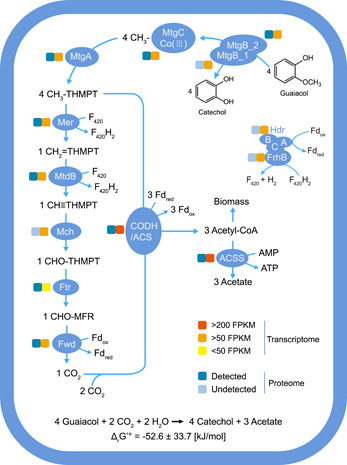
An overview of the central metabolic pathway of *Ca*. Baizosediminiarchaeum ligniniphilus. The process of *O*‐demethylation of guaiacol is accompanied by the fixation of carbon dioxide and the production of acetate. Yellow rectangles of varied shades show different gene transcription levels and blue rectangles of different shades show whether the relevant proteins were detected in the proteomics. Below the pathway is the Gibbs free energy (Δ*G*) estimated for the overall equation. FPKM, fragments per kilo base of transcript per million fragments mapped.

**Figure 4:**
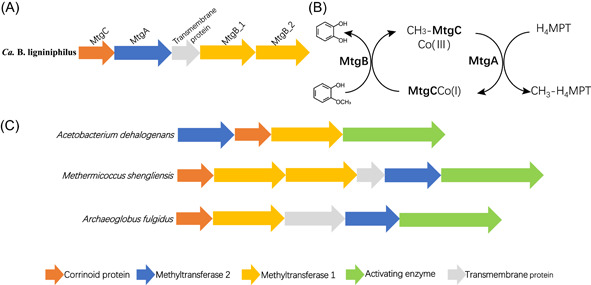
The operon encoding the gene cluster of *O*‐demethylase/methyltransferase complexes. (A) The *O*‐demethylase/methyltransferase gene cluster of *Ca*. Baizosediminiarchaeum ligniniphilus. (B) The methyl‐transfer pathway with guaiacol as the substrate. (C) The gene clusters encoding the *Acetobacterium dehalogenans* vanillate *O*‐demethylase system^8^, *Methermicoccus shengliensis* methoxybenzoate *O*‐demethylase system^11^, and *Archaeoglobus fulgidus* 2‐methoxyphenol *O*‐demethylase system^10^. Co(I) and Co(III), corrinoid protein with cobalt in their respective valence states; H_4_MPT, tetrahydromethanopterin.

One gene cluster that may carry out the *O*‐demethylation/methyltransferase process on ArOCH_3_ was found within the *Ca*. B. ligniniphilus MAG. This putative methyltransferase complex includes two copies of putative methyltransferase 1 (MT1, *mtgB*_1 and *mtgB*_2), one copy of putative methyltransferase 2 (MT2, *mtgA*), and one copy of putative corrinoid protein (CP, *mtgC*) (Figures 3 and 4A). Genes encoding this putative methyltransferase complex of *Ca*. B. ligniniphilus were all transcribed at high levels, with fragments per kilobase of transcript per million fragments mapped (FPKM) values > 70 (Figures 3 and S5). Moreover, proteins of MtgA, MtgB_2, and MtgC were detected in the proteome (Table S3). We proposed that, within this methyltransferase complex, MtgB_1 and MtgB_2 catalyze the demethylation of ArOCH_3_ and transfer the cleaved methyl group to cob(I)alamin bound with MtgC and form CH_3_‐cob(III)alamin. The MtgA catalyzes a subsequent transfer of the methyl group from CH_3_‐cob(III)alamin to H_4_MPT and form cob(I)alamin and CH_3_‐H_4_MPT (Figure 4B).

In the methyltransferase complex, MT1 is the key enzyme responsible for substrate selectivity and determines the type of methyl substrate. The two putative MT1s (MtgB_1 and MtgB_2) of *Ca*. B. ligniniphilus are ~300 amino acids long (~32 kDa). Neither had homology with the known MT1, which indicated that these two MT1s (MtgB_1 and MtgB_2) are novel. MtgB_1 and MtgB_2 shared a protein sequence similarity of 44.85% with each other. This low similarity suggests different substrate affinities for the two proteins, which may target different types of ArOCH_3_.

In this methyltransferase reaction, cobalt switches between oxidation states Co(I) and Co(III). However, because of its low redox potential, the super‐reduced cob(I)alamin is very sensitive to oxidation, which results in the formation of inactive cob(II)alamin. This undesired oxidation product can be reactivated by the AE and converted into the required cob(I)alamin at the expense of ATP. However, the AE was apparently absent or unidentified in the *Ca*. B. ligniniphilus genome (Figure 4A,C). As is the case with MtgB_1 and MtgB_2, the unidentified AE in *Ca*. B. ligniniphilus is likely also novel and requires further identification.

CP (*mtgC*) and MT2 (*mtgA*) of *Ca*. B. ligniniphilus showed homology with the known CP and MT2 lineages from various methyltransferase systems, such as the *M. shengliensis* methoxybenzoate *O*‐demethylase system^11^, *A. dehalogenans* vanillate *O*‐demethylase system^8^, and *Desulfitobacterium hafniense* glycine betaine demethylase system^12^ (Figure S6). *Ca*. B. ligniniphilus likely acquired the CP (*mtgC*) and MT2 (MtgA) for ArOCH_3_ metabolism from other bacteria or archaea through horizontal gene transfer. Additionally, because these methyltransferase systems function in the cytoplasm, their cells require transporters for the uptake of ArOCH_3_. A transmembrane protein within the methyltransferase complex was identified in the *Ca*. B. ligniniphilus genome and considered a candidate transport protein for aromatic compounds (Figure 4A).

A total of five bacterial‐type methyltransferase gene clusters were also found in bacterial MAGs that were coassembled from the enrichment metagenome, but these MAGs showed low relative abundance (<0.5%) (Table S4). Of these, four gene clusters were present at very low expression levels (FPKM < 1.2), and one of them had high expression with FPKM = 9.3–15.0. This gene cluster was extracted from Bin.026, which was classified in the order *Desulfatiglandales* of the phylum *Desulfobacterota*. This suggests that the members of *Desulfatiglandales* might compete with *Ca*. B. ligniniphilus for ArOCH_3_ substrates or might use a different type of ArOCH_3_ in the culture system.

**Figure 5:**
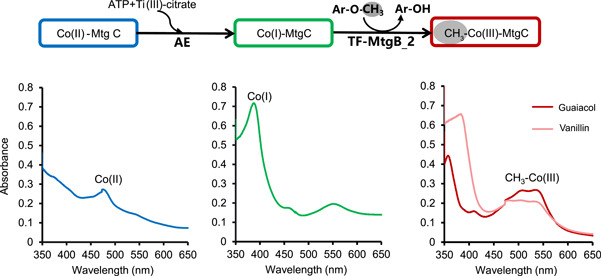
The *O*‐demethylation/methyltransferase activities of *Ca*. B. ligniniphilus with guaiacol and vanillin as the substrates. Reaction 1: Activating enzyme (AE), ATP, and titanium (III) citrate are required for the activation of corrinoid protein (MtgC) from the Co(II) state (blue) to the active Co(I) state (green); AE was from *Acetobacterium dehalogenans* DSM 11527 (GenBank accession no. ACJ01666.1). Reaction 2: MtgB transfers the methyl group from guaiacol to Co(I)‐MtgC, resulting in methylated Co(III)‐MtgC (red and pink). All bottom panels correspond to UV/visible spectra measured after each reaction, reflecting the different states of the cobalamin carried by MtgC. Ti(III), titanium(III).

To verify the function of the novel MT1s of *Ca*. B. ligniniphilus in *O*‐demethylation and methyl transfer, the MtgB_2 (detected in the proteome) and MtgC of *Ca*. B. ligniniphilus were expressed and purified in *Escherichia coli* and subjected to an enzyme activity assay (Figure S7). In addition, for the synthesis of the Co(I) state of CP, an AE gene from the acetogenic bacterium *A. dehalogenans* was also expressed and purified in *E. coli* and included in the enzymatic reaction. First, AE reactivated the Co(II) state by reducing the cobalamin to the active Co(I) state using ATP and titanium(III) citrate, causing a shift of the absorption peak from 480 to 390 nm in the UV‐vis spectrum (Figure 5). Next, the addition of a methoxylated substrate (including guaiacol and vanillin) and MtgB_2 resulted in a shift in the absorption peak from 390 to 520 nm, which indicated the formation of methyl‐Co‐(III), that is, the occurrence of methyl transfer. In addition, the HPLC chromatogram showed a decrease in the guaiacol peak along with an increase in the catechol peak, which further confirmed the occurrence of *O*‐demethylation (Figure S8).

### Metabolic reconstruction of *Ca*. B. ligniniphilus

For the central carbon metabolism of *Bathyarchaeia*, we previously reported that a predecessor slurry enrichment culture containing *Ca*. B. ligniniphilus had incorporated ^13^C‐labeled inorganic carbon (IC) for biomass synthesis and acetate production, with lignin as an energy source^21^. In this study, during the growth of *Ca*. B. ligniniphilus, all genes encoding the pathway of acetogenesis (acetate production involving a CO_2_ reduction step via the Wood–Ljungdahl pathway) were highly transcribed, and most of the pathway proteins were present in the proteome (Figures 3 and S5, Table S3). Moreover, the *Bathyarchaeia*‐specific methyltransferase system was also transcribed and translated. Thus, it is expected that the ArOCH_3_‐derived methyl carried by H_4_MPT would be further oxidized into CO_2_, resulting in the accumulation of reducing equivalents distributed among multiple electron carriers (e.g., in the forms of reduced ferredoxin and F_420_H_2_). *Ca*. B. ligniniphilus is also capable of converting the methyl group carried by H_4_MPT into acetyl‐coenzyme A (CoA) by the addition of CO_2_ that would consume reduced ferredoxin. Furthermore, the produced acetyl‐CoA can be used for biosynthesis and acetate production. The coenzyme F_420_ hydrogenase subunit beta (FrhB) and heterodisulfide reductase (HdrABC) could mediate the conversion of reducing equivalents between ferredoxin and F_420_. Such recycling of reducing equivalents has also been observed in acetogenic bacteria and methanogenic archaea that conduct CO_2_‐reducing acetogenesis/methanogenesis, which allows cells to reoxidize excess reducing power produced from oxidation of ArOCH_3_‐derived methyl groups^11^, ^24^, ^25^.

With guaiacol as the substrate, the overall reaction yielded a Gibbs free energy of −52.6 ± 33.7 kJ/mol (Figure 3). This low energy yield provides an explanation for the slow growth of *Ca*. B. ligniniphilus (Figure 1B). In addition, from 60 days to 120 days of culture, the number of *Ca*. B. ligniniphilus cells doubled (Figure 1C); however, all detectable ArOCH_3_ were almost depleted at 30 days (Table 1). This suggests that the growth of *Ca*. B. ligniniphilus might not rely on ArOCH_3_ as a substrate at this stage, but they may use organic matter produced by their own cells or other microbial cells in the culture system.

### Distribution of the methyltransferase in bathyarchaeial MAGs and in situ coastal sediments

In a previous study, one MAG of *Bathyarchaeia* was also reported to carry a bacterial‐type methyltransferase system, and these genes were acquired by horizontal gene transfer^10^, ^11^. In this study, public genomic data were extensively searched for genes coding for the bacterial‐type methyltransferase system. We found that 31 out of 297 analyzed bathyarchaeial MAGs contained the bacterial‐type methyltransferase gene cluster or the bacterial‐type methyltransferase 1 (MT1) gene. These MAGs were distributed in five of the eight orders of *Bathyarchaeia* (Figure 2 and Table S5). The *Ca*. B. ligniniphilus genome contained the two copies of putative MT1s (*mtgB*_1 and *mtgB*_2) instead of bacterial‐type MT1. We found that, unlike the genes of bacterial‐type MT1, which are widely detected in both bacterial and archaeal lineages and shared by horizontal gene transfer, the genes encoding MtgB were only found in the genomes of *Bathyarchaeia*.

In 297 bathyarchaeial MAGs, 60 MAGs were found to contain the *Bathyarchaeia*‐specific *mtgB* gene or its gene cluster; intriguingly, all 60 MAGs clustered in the order *Baizomonadales* ^22^, ^23^ (Figure 2 and Table S5). Thus, the evolution model of *Bathyarchaeia*‐specific *mtgB* genes may be vertical inheritance because they likely evolved from the common ancestor of the order *Baizomonadales*. These homologous MtgB enzymes clustered into three groups based on phylogenetic analysis (Figure S9). The MtgB_1 and MtgB_2 of *Ca*. B. ligniniphilus clustered within groups 1 and 2, respectively. Because group 3 showed a long evolutionary distance from the former two groups, it is unclear whether group 3 would retain the same function. *Bathyarchaeia*‐specific MtgB may be more efficient compared with bacterial‐type MT1, resulting in the predominance of *Ca*. B. ligniniphilus in the lignin culture system (Figure 1A).

**Figure 6:**
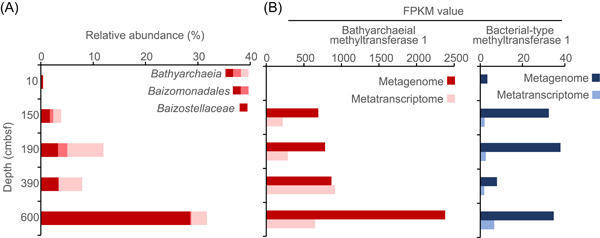
Distribution of the *Bathyarchaeia*‐specific methyltransferase 1 (MT1, *mtgB*) genes in coastal sediments from the East China Sea. (A) The relative abundance of *Bathyarchaeia*. (B) The gene abundance and gene expression level of *Bathyarchaeia*‐specific MT1 (red) and bacterial‐type MT1 (blue).

*Ca*. B. ligniniphilus belongs to the formerly assigned subgroup Bathy‐8 and is currently assigned to the family *Baizostellaceae* in the order *Baizomonadales* ^20^, ^23^. Bathy‐8 (or family *Baizostellaceae*) is frequently detected in high abundances in coastal sediments, especially in deep sediment layers with low redox potential, and lacks electron acceptors^16^, ^19^, ^26^, ^27^, ^28^. In this study, we collected a 600‐cm gravity core of coastal sediment from the East China Sea. After metagenomic and metatranscriptomic analyses, we found that the members of the family *Baizostellaceae* showed high relative abundance (28.5%) in the bottom layer (600 cm) (Figure 6A). The genes of *Bathyarchaeia*‐specific MT1 were present in high abundance in 600 cm, with the FPKM = 2379, which was 68 times greater than that of bacterial‐type MT1 genes (Figure 6B). The expression levels of *Bathyarchaeia*‐specific MT1 genes were also high, with FPKM = 649.5 in 600 cm, which was two orders of magnitude greater than that of the bacterial‐type MT1 genes (Figure 6B). In addition, we found that the *Bathyarchaeia*‐specific MT1 genes were widely distributed in coastal sediments around the world (Table S6). Thus, *O*‐demethylation from ArOCH_3_ catalyzed by these *Bathyarchaeia*‐specific methyltransferase systems likely constitutes a vital step in the anaerobic degradation of lignin in coastal sediments.

More than half (172/297) of the bathyarchaeial MAGs belonged to the order *Baizomonadales*, which are phylogenetically diverse (Figure 2 and Table S5)^20^, ^22^, ^23^. Additionally, more than a third of the MAGs (60/172) in the order *Baizomonadales* contained the *mtgB* gene or its gene cluster; these MAGs were assembled from diverse anoxic habitats, including termite guts, mangrove sediments, subsurface fracture fluids, permafrost active layer soils, marine sediments, peatlands, peat soils, estuary sediments, hot spring sediments, and mud volcanos. This suggests that the order *Baizomonadales* may play an important role in *O*‐demethylation from ArOCH_3_ in broad anoxic environments, and that MtgB may confer fitness advantages to the order *Baizomonadales*, which would allow this lineage to become ubiquitous and the most representative bathyarchaeial lineage.

Marine sediment is one of the largest organic carbon reservoirs on the Earth, with coastal sediment (water depth <200 m) contributing >50% of the total organic carbon buried in marine sediments^29^. In coastal sediments with high organic matter loading, oxygen is quickly exhausted within the top few millimeters to centimeters^30^, ^31^; this produces an extensive anoxic zone where a large proportion of organic carbon persists, including a considerable amount of recalcitrant lignin^32^, ^33^, ^34^. In this study, we achieved a continuous laboratory enrichment of the ubiquitous *Bathyarchaeia* from coastal sediments. This bathyarchaeial archaeon uses a novel methyltransferase system for transferring methyl from lignin‐derived ArOCH_3_. The system is specific to *Bathyarchaeia* and is particularly prevalent in the order *Baizomonadales*. Given the high abundance and widespread distribution of *Baizomonadales* and their methyltransferase in anoxic coastal sediments, demethylation catalyzed by their *Bathyarchaeia*‐specific methyltransferase system is likely a critical step in anaerobic lignin degradation.

## MATERIALS AND METHODS

### Etymology

We propose the candidate name “*Ca*. Baizosediminiarchaeum ligniniphilus” DL1YTT001 for this bathyarchaeial member. Etymology: Baizosediminiarchaeum, Bai.zo.se.di.mi.ni.ar.chae'um. N.L masc. n. Bai Ze, a propitious white creature living in the cold Kunlun Mountains in Chinese mythology; L. neut. n. sedimen ‐inis, sediment; N.L. neut. n. archaeum, ancient one, archaeon; N.L. neut. n. Baizosediminiarchaeum, an archaeon from sediment and named after Baize, a propitious creature in Chinese mythology. Ligniniphilus, lig.ni.ni'phi.lus. N.L. neut. n. ligninum, lignin; N.L. adj. philus ‐a ‐um, friend, loving; from Gr. adj. philos ‐ê ‐on, loving; N.L. masc. adj. ligniniphilus, lignin‐loving, isolated as a lignin degrader with lignin as the single organic carbon source.

### Sample collection

Sediment samples for cultivation were collected from an intertidal zone (30.592817 N, 122.083493 E) on Dayangshan Island in Hangzhou Bay of the East China Sea^21^. Samples were placed in oxygen‐free gas‐tight bags and stored at 4°C until further culture experiments. A gravity corer was used in the East China Sea (31.104444 N, 122.576111 E) for sediment sample collection, and samples were stored at −80°C for further metagenomic and metatranscriptomic sequencing.

### Cultivation conditions

Initial cultivation setup of the intertidal sediments has been previously described^21^. Routine transfers were performed at 2–3‐month intervals using a sulfate‐free artificial seawater medium supplemented with 50 mM NaHCO_3_ and 5 g/l alkali lignin (CAS:8068‐05‐1; Sigma). To purify *Ca*. B. ligniniphilus, the enrichment culture was supplemented with mixed bacteria‐specific antibiotics (ampicillin, vancomycin, kanamycin, and streptomycin, each at a concentration of 50 µg/ml), as well as an additive of the supernatant (1:10) filtered (pore size, 0.22 μm) from the enrichment culture without adding an antibiotic (Figure S4).

### DNA and RNA extraction, and metagenomic and metatranscriptomic sequencing

DNA extraction for 16S rRNA gene amplification was performed using the PowerSoil DNA Isolation Kit (Qiagen). Total DNA for metagenome sequencing was obtained using an Advanced Soil DNA Kit (mCHIP). Total RNA was recovered from the 1‐month‐old culture using an RNeasy® PowerSoil® Kit (Qiagen). The kits mentioned above were used according to the manufacturer's protocols.

Libraries for metagenomic sequencing were generated using the NEB Next® Ultra™ DNA Library Prep Kit for Illumina® (New England Biolabs) following the manufacturer's recommendations with the addition of index barcodes. For metatranscriptomic sequencing, the detailed methods involving the removal of rRNA and library construction are described in the Supplementary Methods. The metagenomic and metatranscriptomic libraries were sequenced on an Illumina Novaseq. 6000 platform (Illumina) and generated 150‐bp paired‐end reads.

### qPCR

Bacterial and bathyarchaeotal 16S rRNA genes were quantified by qPCR using the primer pairs Bac341F/prokaryotic519R^35^ and Bathy‐442F/Bathy‐644R^28^, respectively, according to a previously described method^28^.

### 16S rRNA gene amplification and analysis

The hypervariable V4 region of the prokaryotic 16S rRNA genes was amplified using the primer set 515F–806R^36^. Sequence reads were obtained from the Illumina NovaSeq platform based on 2 × 250‐bp cycles and the NovaSeq. 6000 SP Reagent Kit (500 cycles; Illumina). Further bioinformatic analysis was performed using the QIIME 2 standard pipeline (v2020.11)^37^.

### Metagenomic assembly, binning, and annotation

Trimmomatic (v0.38) was used to remove possible adaptors and low‐quality bases for each read^38^. The dereplicated, trimmed, and paired‐end DNA reads were assembled using MEGAHIT^39^ and SPAdes De Novo Assembler^40^. For assembled contigs longer than 1 kb, open reading frames were predicted and translated using prodigal (v2.6.3) with ‐p meta parameters^41^. Clean reads were mapped onto their assembled contigs using bowtie2 (v2.2.8) with very sensitive mode^42^. Assembled contigs (longer than 1 kb) were binned into putative taxonomic groups based on abundance information using MaxBin (v2.2.4)^43^. Detailed estimates of genome contamination and completeness were assessed based on lineage‐specific marker sets using CheckM^44^. The retrieved MAGs were annotated using eggnog‐mapper‐1.03 in the EggNOG database with an e‐value of 10^−10^.

The completeness of specific pathways and functions within the MAGs was assessed based on the canonical pathways available in the KEGG Pathway Database (www.kegg.jp). Relative abundances of all the MAGs were calculated using CoverM (v0.6.1; https://github.com/wwood/CoverM). Unmapped reads were removed for further analyses.

### Metatranscriptomic analysis

For metatranscriptomic analysis, sequence reads were filtered to remove low‐quality and rRNA reads and then mapped to metagenome‐assembled contigs using TopHat (v2.1.1)^45^. To estimate the expression level of each gene, the expected FPKM values were calculated using Cufflinks (v2.2.1) (http://cole-trapnell-lab.github.io/cufflinks/).

### Phylogenetic analyses

A total of 296 representative bathyarchaeial MAGs (Table S5) and nine archaeal MAGs of the class *Nitrososphaeria* were downloaded from the following genome databases: NCBI (https://www.ncbi.nlm.nih.gov/), GTDB (Genome Taxonomy Database)^22^, and the GEM (Genome from Earth's Microbiome) catalog^46^. These MAGs were used to construct the phylogenomic tree with the *Ca*. B. ligniniphilus MAG (DL1YTT001) from this study. The phylogenomic tree was constructed based on a concatenated alignment of a set of 37 marker genes^47^, ^48^ using the MAFFT^49^ algorithm (v7.313) and filtered using trimAl^50^ (v1.4.rev2). Then, the phylogenetic trees were built using both IQ‐Tree^51^ (v1.6.6) and RAxML52^52^ (v8.0) with a bootstrap value of 1000. iTOL (http://itol.embl.de/) was used to modify the phylogenetic trees.

### Proteomic analysis

After 7 days of cultivation, cell pellets were harvested under anoxic conditions from 10 ml of culture by centrifugation at 13,000*g* for 25 min, frozen in liquid nitrogen, and stored at −80°C. The protein of cell pellets was extracted for LC‐MS/MS analysis using an Easy nLC1200/Q Exactive plus mass spectrometer. The detailed sample preparation and analytical method using LC‐MS/MS is described in the Supplementary Methods.

### GC‐MS analysis of LMW aromatic compounds

GC‐MS was used to monitor LMW aromatic compounds during incubation. Basic experimental procedures were carried out following the methodology modified from Raj et al.^53^ Specifically, 2‐ml aliquots of the enrichment cultures or controls were sampled at 0 and 30 days. LMW aromatic compounds were extracted and then identified and quantified on a Trace 1310 gas chromatograph coupled to a TSQ8000 mass spectrometer (Thermo Fisher Scientific) using an HP‐5MS capillary column (30 m × 0.25 mm i.d., 0.25‐μm film thickness). The detailed sample preparation and analysis using GC–MS are described in the Supplementary Methods.

### Heterologous protein production of MtgC and MtgB

The genes encoding the methyltransferase I (MtgB_2) and corrinoid protein (MtgC) were amplified from the culture samples for cloning in expression vectors pCold‐TF and pET‐28a, respectively. For Co(I) production, the AE gene of *A. dehalogenans* DSM 11527 (GenBank accession no. ACJ01666.1) was synthesized and then amplified for cloning in expression vector pET‐28a. For production of MtgB_2, MtgC, and AE, the plasmids were used for transformation into *E. coli* BL21 (DE3). The detailed method of heterologous protein production is described in the Supplementary Methods.

### Enzyme activity assays

The enzyme activity assays were adapted from Schilhabel et al.^8^ and Kurth et al.^11^ and were performed in 350‐µl anaerobic Quartz cuvettes (Purshee Co., Ltd.) that were sealed with rubber stoppers and gassed with N_2_. MtgB protein activity was determined in 300 µl of buffer containing 35 mM Tris–HCl (pH 7.5) and 70 mM KCl. First, reconstituted Co(II)‐MtgC at a final concentration of 1.2 mg/ml (~79 µM) was activated by adding 12 mM MgCl_2_, 0.5 mM Ti(III) citrate (freshly prepared), and 2.3 mM ATP. The conversion to Co(I)‐MtgC was followed by a change in absorbance at 387 nm on a HACH device (spectrophotometer, DR/5000 Company). The enzymatic reaction was started by the addition of 2.3 mM guaiacol and vanillin, and MtgB at a final concentration of 0.015 mg/ml. Fifty microliters of the sample were collected before and after the enzymatic reaction to analyze the methoxy aromatic compounds and products using HPLC (see Supplementary Methods). Additionally, the formation of CH_3_‐Co(III)‐MtgC from Co(I)‐MtgC was expected to result in a decrease in absorption at 387 nm and an increase in absorption at 530 nm on a spectrophotometer. As a negative control, 2.3 mM methanol was used.

## AUTHOR CONTRIBUTIONS

**Tiantian Yu**: Conceptualization (equal); data curation (equal); formal analysis (lead); investigation (equal); methodology (lead); project administration (equal); writing—original draft (lead); writing—review and editing (equal). **Haining Hu**: Data curation (equal); methodology (equal); writing—review and editing (supporting). **Xianhong Zeng**: Data curation (equal); methodology (equal). **Yinzhao Wang**: Writing—review and editing (supporting). **Donald Pan**: Writing—review and editing (supporting). **Longhui Deng**: Writing—review and editing (supporting). **Lewen Liang**: Data curation (supporting). **Jialin Hou**: Data curation (supporting). **Fengping Wang**: Conceptualization (lead); funding acquisition (lead); project administration (lead); writing—review and editing (lead).

## ETHICS STATEMENT

No animals or humans were involved in this study.

## CONFLICT OF INTERESTS

The authors declare no conflict of interests.

## Supporting information

Supporting information.

Supporting information.

## Data Availability

The MAGs reported in this paper and illumina sequencing raw data of 16S rRNA gene amplicon sequences were submitted to NCBI under accession numbers PRJNA983078 and PRJNA983059, respectively.
